# Hospital Resource Planning for Mass Casualty Incidents: Limitations for Coping with Multiple Injured Patients

**DOI:** 10.3390/healthcare11202713

**Published:** 2023-10-11

**Authors:** Daniel Staribacher, Marion Sabine Rauner, Helmut Niessner

**Affiliations:** 1 Medical University Vienna, Spitalgasse 23, A-1090 Vienna, Austria; daniel.staribacher@meduniwien.ac.at; 2 Clinic for Neurosurgery, Sozialstiftung Bamberg, Buger Straße 80, D-96049 Bamberg, Germany; 3 Department of Business Decisions and Analytics, Faculty of Business, Economics, and Statistics, University of Vienna, Oskar-Morgen-Stern-Platz 1, A-1090 Vienna, Austria; 4 SimPlan Optimizations e. U., Ilse-Arlt-Straße 12/161, A-1220 Vienna, Austria; office@helmutniessner.at

**Keywords:** discrete-event simulation, simulation-optimization, multiple regression, disaster management, mass casualty incidents, triage, resource allocation, trauma patient treatment, hospital

## Abstract

Using a discrete-event simulation (DES) model, the current disaster plan regarding the allocation of multiple injured patients from a mass casualty incident was evaluated for an acute specialty hospital in Vienna, Austria. With the current resources available, the results showed that the number of severely injured patients currently assigned might have to wait longer than the medically justifiable limit for lifesaving surgery. Furthermore, policy scenarios of increasing staff and/or equipment did not lead to a sufficient improvement of this outcome measure. However, the mean target waiting time for critical treatment of moderately injured patients could be met under all policy scenarios. Using simulation-optimization, an optimal staff-mix could be found for an illustrative policy scenario. In addition, a multiple regression model of simulated staff-mix policy scenarios identified staff categories (number of radiologists and rotation physicians) with the highest impact on waiting time and survival. In the short term, the current hospital disaster plan should consider reducing the number of severely injured patients to be treated. In the long term, we would recommend expanding hospital capacity—in terms of both structural and human resources as well as improving regional disaster planning. Policymakers should also consider the limitations of this study when applying these insights to different areas or circumstances.

## 1. Introduction

Mass casualty incidents (e.g., accidents in mass transportation, terrorist attacks, natural disasters) are rare events in which a large number of people with urgent medical needs can quickly overwhelm the existing capacities of available healthcare [1,2]. As such events usually happen without warning, disaster management is of critical importance for policymakers to prevent them, to mitigate the potential harm, and to prepare for them in order to ensure the best possible response, as well as to facilitate a rapid recovery so as to learn how to better prepare for future events [2,3,4]. Resilience in healthcare [5] plays an essential role, especially in times of cascading disasters, such as an earthquake followed by a tsunami [6]. Therefore, emergency plans that are sound and robust are urgently needed in healthcare [7].

Over the past few years, many hospitals have prepared emergency plans for intensive care units (ICUs) and emergency departments to better manage the allocation of scarce resources in the event of mass casualty incidents [8,9]. Most commonly, real on-site hospital exercises have been used to investigate the readiness and adequacy of available resources and to optimize the underlying strategies applied. This approach has drawbacks; it is expensive in terms of opportunity costs, and its findings are not reproducible [10]. By contrast, Operations Research can provide a wide range of qualitative and quantitative methods to support replicable and affordable decision-making in healthcare to foster resilience in the case of disasters [5,11]. By using scenario analysis and sensitivity analysis, a variety of different policy situations with unknown parameters and diverse circumstances can be investigated and evaluated based on key metrics, such as costs, efficiency, and effectiveness [12].

For complex and dynamic resource allocation, simulation-based approaches are especially suitable for visualization-based decision-making and, therefore, have been accepted in healthcare practice for many years [13,14,15]. Recently, hybrid simulation modeling has gained importance [16]. In addition, simulation-optimization techniques [17] can further augment and improve decision-making.

By using advanced simulation techniques [16,17], healthcare organizations, such as hospitals, can identify relevant bottlenecks, such as the number of staff members, machines, vehicles, equipment, material, and space. The impact of legal, political, social, economic, or other frameworks can also be investigated on outcome measures. For example, agent-based simulation allows for analyzing the behavior of healthcare stakeholders, such as patients, staff members, healthcare managers, suppliers, and social insurance companies [18].

Consequently, simulation-based approaches have been used successfully to improve preparedness and decision-making regarding mass casualty events from incident sites over ambulance services to hospitals [9,19,20]. For example, Debacker et al., 2016 [21] implemented a comprehensive discrete-event simulation-based disaster medical response system to better respond to airplane crashes at an international airport. Next, disaster management games can be used to train ambulance service staff on various priority rules to triage patients for treatment at the incident site based on simulation-optimization approaches [22,23,24]. In addition, Tallach et al., 2022 [25] applied simulation techniques to improve resilience by identifying latent design flaws and safety issues for hospitals. Then, Chen 2018 [26] found that computer tomography scanners were the bottleneck that led to delays in one hospital’s treatment of mass casualty incidents.

The aim of this study for managing mass casualty incidents was to identify potential bottlenecks and provide a realistic performance indicator, which is the number of multiple injured patients that can feasibly be treated in a major acute specialty hospital located in Vienna, Austria. The time until critical treatment of these casualties should also be kept within a medically acceptable time frame, which can also be determined by using discrete-event simulation.

Furthermore, we also used a simulation-optimization approach to support decision-makers in order to enhance the number of severely injured patients that can be treated and by correspondingly reducing their mean total waiting time for critical treatment. In the short run, this can be achieved by increasing the number of medical staff and/by optimizing the staff-mix. We also applied a multiple regression model to identify key staff categories to shorten the mean total system time and to improve the survival of patients with multiple injuries. Section 2 outlines the purpose, material, and methods of the study, while Section 3 presents the results. The paper closes with a discussion (Section 4) and a conclusion (Section 5).

## 2. Purpose, Material, and Methods of the Study

Our aim was to thoroughly investigate the resource management for severely and moderately injured patients following a mass casualty incident at an acute specialty hospital in Vienna, Austria. We applied discrete-event simulation at the micro (patient) level in order to determine how this specialty hospital would handle such an extreme situation [13,14,15,16]. In addition, we examined whether the requirements of the underlying hospital disaster plan were feasible and manageable regarding the allocation of 5 severely, 20 moderately, and 50 slightly injured patients [27].

We additionally focused on disclosing bottlenecks and limitations regarding resources (e.g., equipment, material, staff, and space). Furthermore, we analyzed whether optimized simulated staff-mix strategies for this hospital could enhance the handling of mass casualty incidents. This study also aimed to support regional policymakers to better manage the allocation of patients among Viennese hospitals in the future.

The simulation was created using the simulation software AnyLogic 7 & 8 [28,29]. This software tool allows modeling and visualizing complex decision processes for advanced decision support by applying different types of simulation approaches (discrete-event simulation, agent-based simulation, system dynamics) combined with optimization techniques simultaneously (either the Opt-Quest optimization tool or own optimization approaches implemented in JAVA). For this study, we used the Opt-Quest optimization tool of the simulation software AnyLogic [29]. This tool integrates state-of-the-art meta-heuristic procedures, including tabu search, neural networks, scatter search, and linear/integer programming, into a single composite method. Using simulation-optimization, we investigated an optimal staff-mix for an illustrative policy scenario in our study. Finally, multiple regression analyses were applied to the enumerated simulated staff-mix policy scenarios to reveal the staff categories with the highest impact on critical outcome measures.

The original basic simulation model was created and calibrated by [27]. It was based on the knowledge gained by past discrete event simulation studies on disaster management [22,23] as well as unpublished simulation studies in Viennese hospitals by one of the other authors. Furthermore, we applied our experience of a past simulation-optimization study by [24], a disaster management game handling mass casualty incident on site by ambulance rescue services. From this past study, we took the triage and survival concepts of patients described in detail in Section 2.2.1. In this past study, we also demonstrated that the Opt-Quest optimization tool of the simulation software AnyLogic performed very well compared to other simulation-optimization approaches [17]. Therefore, we again applied this Opt-Quest optimization tool in the current study presented, which was not included in [27]. In addition, the multiple regression analyses were not part of [27].

In Section 2.1, we present details on the decision support system of the simulation-optimization study, including input variables, outcome measures, and policy scenarios, while the underlying data are described in Section 2.2.

### 2.1. Simulation-Optimization Study

The study is based on an acute specialty not-for-profit hospital in Vienna, Austria, with around 450 beds run by a social insurance company [27]. It contains the following departments: internal medicine with three intermediate care beds with ventilation capacity; anesthesiology with 10 intensive care beds with ventilation capacity; ophthalmology; general surgery; gynecology; ear, nose, and throat (ENT); trauma surgery; and urology. Furthermore, a central laboratory and a central radiology institute are available on-site.

The simulation study for this hospital starts with the triage of patients at the entrance of the trauma surgery emergency department in case of a mass casualty incident. Patients are triaged to a specific treatment category [30]: (1) slightly injured (green), (2) moderately injured (yellow), and (3) severely injured (red). The health status of the patients is modeled by a scoring system that improves with prompt treatment and deteriorates at varying rates based on the time until treatment (ranging from perfect health = 99 to death = 0). In that case, deceased patients (black) are assigned to an absorbing state (and are collected in special rooms). A similar approach was also used for an ambulance service management disaster game [22].

Triage is a process of allocating limited resources to specific patients in order to offer the greatest possible benefit to the greatest number of patients. The overall goal of the triage process is to maximize the number of lives saved. There are many different triage systems in use worldwide [30]. All of them have in common that they quickly assess the priority for the treatment of patients related to their needed resources. In this acute specialized hospital, a tag with color coding is used to allocate a patient to a specific triage category. Within each triage category, there is usually no further (sub-)triage, and a first come, first serve policy is applied. In case of a mass casualty incident, it is not predictable how many patients will be triaged to a given category. Subsequently, treatment of patients according to the triage category is delivered as quickly as possible.

In this study, changes in the triage category were accounted for based on [22]. Known as “progressive patient care”, if a patient were triaged in an urgent category and her health improved, she would then be triaged into a different category without the need for urgent care. However, for example, if the health of a moderately injured patient greatly deteriorated, his new triage category would reflect his severity and also his risk of mortality.

Severely injured patients (triaged red) are brought to the resuscitation area or a second improvised resuscitation area in the procedures room. Moderately injured patients (triaged yellow) are generally treated in the acute outpatient department or in the cast room. However, if moderately injured patients are waiting too long for treatment, their health may worsen. Thus, their status may change to ”red”, and they might further be assigned to the related rooms for severely injured patients. Slightly injured patients (triaged green) need no further acute care treatment and are gathered in the dining hall, where they await discharge. Moderately and severely injured patients might need further treatment or interventions at the following locations:

- X-ray examination rooms;
- Computed tomography imaging rooms;
- Operating rooms.

Afterward, these moderately or severely injured patients are hospitalized. The hospitalization of these patients in general medicine or ICU departments is not further considered in the simulation. We assume that the general medicine departments of this hospital have sufficient capacity or can free up capacities for these patients. In the ICU of this specialized hospital, capacities can also be freed up, or—if not—these patients might be transferred to nearby hospitals in Vienna, which is a realistic scenario for mass casualty incidents.

**Figure 1:**
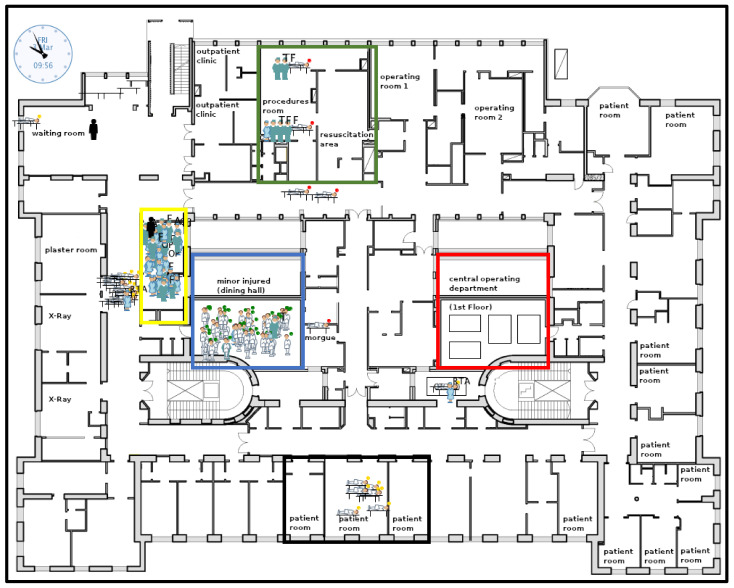
Simulation interface for handling mass casualty incidents in an acute specialty hospital in Vienna, Austria (visualization of the baseline scenario #1). **Patients:** standing (green point: slightly injured), laying in beds (yellow point: moderately injured; red point: severely injured). **Medical and non-medical staff:** physicians, nurses, radiology assistants, and surgical assistants. **Black box:** patient room. **Blue box:** dining hall. **Green box:** first aid room and shock room. **Red box:** ICU including operating rooms. **Yellow box:** common room.

Figure 1 displays the visualization of the trauma surgery emergency department on the ground floor (e.g., waiting areas, examination rooms, operating rooms), the intensive care unit with additional operating rooms on the first floor (red box), and the dining hall in the basement (blue box). Medical and non-medical staff are visualized according to their category, are gathered in the common room (yellow box) and are assigned to varying treatment rooms or varying duties. Slightly injured patients (indicated with a green point in standing positions) are gathered in the dining hall of the hospital (blue box). For example, moderately injured patients are indicated with a yellow point lying in beds, such as in the patient room with six beds (black box). Severely injured patients indicated with a red point lying in beds are treated in the procedures room by medical doctors and nurses (green box).

#### 2.1.1. Input Variables

To investigate how this specialty hospital can cope with its limited resources for different severe mass casualty policy scenarios, the following key patient-related input variables can be varied in the simulation (arrival rates were based on exponential distributions):

- Number of severely injured patients;
- Number of moderately injured patients;
- Number of slightly injured patients;
- Time span in which the patients above arrive.

Furthermore, policymakers can analyze the impact of key limited human and non-human resources to successfully master the impact of different severe mass casualty incidents by altering the following key hospital-related input variables in the simulation:

- Number of operating rooms;
- Number of radiology machines/computer tomography machines;
- Number of key staff members and their mix (e.g., physicians: medical specialists, medical assistants, rotation physicians; nurses: general nurses, surgical nurses; radiology assistants; others: surgical assistants).

Finally, the following input variables were established for the simulation of the current disaster plan for this hospital:

- Ratio of slightly injured patients per nurse (1:10);
- Ratio of slightly injured patients per rotation physician (1:25);
- Time of day for the mass casualty incident (10 p.m.).

#### 2.1.2. Outcome Measures

Outcome measures are essential for policymakers to analyze how this specialty hospital can cope with varying mass casualty incident scenarios. Therefore, the following outcome measures are provided by the simulation:

- The workload of staff members by key category (e.g., physicians: medical specialists, medical assistants, rotation physicians; nurses: general nurses, surgical nurses; radiology assistants; others: surgical assistants);
- The utilization of non-human resources (e.g., X-ray machines, computer tomography machines, operating rooms, resuscitation areas, transport beds);
- The mean waiting time of patients by triage category.

The most critical outcome measure for policymakers in the field of mass casualty events comprises the mean waiting time of injured patients for treatment and especially the mean waiting time for a surgical intervention, which are closely related to patient outcome (survival, disease progression, and long-term care) [31]. Currently, such waiting time guidelines do not exist in the field of trauma care. However, several waiting time benchmarks for time-critical surgeries of moderately and severely injured patients could be used as suitable benchmarks for the time span between the arrival of the patient at the hospital and the first initial treatment (for non-surgery patients) or surgery (for surgical patients). Thus, this time span was used as the key outcome measure for our simulation entitled “mean waiting time for critical treatment” in the results Section 3. Please note that moderately and severely injured patients have different urgencies to be treated.

Thus, for moderately injured patients, the time from arrival at the hospital to the beginning of surgery should be less than 12 h (720 min), which was derived from the current recommendation for the treatment of time-critical injuries, such as open fractures [32]. Thus, the benchmark of 720 min was selected for moderately injured patients as a suitable medical boundary for the outcome variable “mean waiting time for critical treatment”.

For severely injured patients, the time from arrival at the hospital to the beginning of a surgical procedure should not exceed 120 min because, between about 120 and 180 min after hospitalization, the highest incidence of deaths from bleeding occurs [33]. Since surgical hemostasis is the treatment of choice in the case of an acutely bleeding patient, this surgery benchmark of 120 min was chosen for severely injured patients as a suitable medical boundary for the outcome variable “mean waiting time for critical treatment” [31].

One could argue that the percentage of patients whose waiting time exceeds a critical threshold would be a better key performance measure; in this case, one would lose the information on the current “mean waiting time for critical treatment” of moderately and severely injured patients and slackens the critical boundaries of 720 and 120 min, respectively. When developing the policy scenarios in Section 2.1.3, we investigated a variety of other performance indicators and reported the most interesting findings to the policymakers. However, depending on the policymakers and their interest, several additional outcome measures can be displayed or analyzed.

#### 2.1.3. Policy Scenarios

To analyze the influence of the key input variables (cf. Section 2.2.1: e.g., patient-related input variables, hospital-related input variables) on the results of the simulation model, we first performed one-way sensitivity analyses. For the most promising scenarios, we then performed combinations of several two-way and more-way sensitivity analyses to explore the entire solution space. The most interesting effects and results are illustrated by the different policy scenarios presented to evaluate the current disaster plan of this hospital, identify bottlenecks, and disclose boundaries:

1. Impact of the arrival time of injured patients (cf. Section 3.1.1);
2. Impact of the number of injured patients (cf. Section 3.1.2);
3. Impact of the number of staff available (cf. Section 3.1.3);
4. Impact of the number of computer tomography machines (cf. Section 3.1.4).

### 2.2. Data

The input data were based on an existing disaster plan of this hospital before the COVID (Coronavirus disease)-19 pandemic [27], assuming the least available human resources (Section 2.2.1); main equipment, structural and construction resources (Section 2.2.2); instruments and material requirements (Section 2.2.3); as well as patient data (Section 2.2.4).

#### 2.2.1. Human Resources

At least one and up to three specialized physicians are present, supported by up to two assistant physicians depending on the department and medical specialty. In the departments with beds, there are generally two nursing staff members on night duty, at least one of whom is a qualified nurse [27]. The first internal medicine department, as well as the anesthesiology department, require more nursing staff members. In the respective surgical areas, there are also nurses with special training in surgery on duty. During the night, at least two radiology assistants and one radiologist are on shift. Furthermore, at least one porter, two cleaners, and one on-call technician are available in the building around the clock.

The insight from on-site past disaster exercises was that the transport or pick-up and delivery service responsible for transporting patients and material within the hospital might be a bottleneck [27]. Currently, the staff of the external ambulance service takes the patients to the triage of the hospital’s trauma surgery unit. From there, severely injured patients (red) are transported by surgical assistants to the shock room(s). Radiology assistants take non-severely injured patients who are unable to walk to the radiology rooms. Further transportation of patients is partly performed by treating staff. For our mass casualty policy scenarios, transportation of patients was not a bottleneck and thus not further investigated in detail.

In case of mass casualties, the radiology department might switch from computer tomography examinations to the more readily available X-rays or ultrasound examinations for eligible patients to increase capacity for emergencies [34].

To conclude, there might be staff shortages at various levels in the event of a disaster or a mass casualty incident. Some of them can at least be reduced by more time-saving procedures. Others, such as the surgical treatment of patients, pose greater challenges to resource planning. This immediately raises the question of the recruitability of off-duty personnel. This planning is extremely difficult since it is not possible to predict how many staff members can actually be called into service from their free time and in what period of time. Studies found that between 18% and 84% of the hospital employees reached would actually show up [35]. Therefore, one has to account for sufficient lead time since off-duty staff members are unlikely to be available in the first phase of a disaster, especially in the first 1 to 2 h. This is why we only took into account the available staff on duty for mass casualty incidents, as the handling of such events normally takes a couple of hours.

However, for disaster planning with a longer time horizon, this additional off-duty staff should be considered as well. In addition, other suitable staff such as staff on maternity leave or training, former staff members (e.g., people retired, people left), or military/civil servants might be considered for longer-lasting disasters such as epidemics/pandemics. For example, to fight the COVID-19 pandemic, staff members from all of those possible suitable staff categories were acquired worldwide [36].

#### 2.2.2. Equipment as Well as Structural and Constructional Resources

In the case of a disaster, the expansion of equipment as well as structural and construction resources in the short term is even more difficult than the increase in human resources. Recommendations for equipment, as well as structural and constructional resources, are given in the German S3 guideline for treating polytrauma/severe injuries [37], including guidelines for hospital layout, helicopter landing sites, treatment rooms, radiology, laboratories, ventilators, and monitoring machines. Currently, the hospital runs one such certified trauma room.

For example, regarding equipment, the number of big-ticket technologies (e.g., computer tomography machines, magnetic resonance imaging machines) can only be increased in the medium term, if at all, because of tight budgets or regional limitations for their allocation among public hospitals in Austria [38,39]. The number of stretchers was not a bottleneck for our mass casualty incident policy scenarios and was, therefore, not simulated as a critical resource.

Constructional resources (e.g., number of examinations, treatment, monitoring, and operating rooms) in hospitals, on the other hand, can hardly be increased for acute care in the appropriate amount of time. Merely a misappropriation is conceivable and also necessary in the case of this hospital. The current disaster plan of this hospital allows for the misappropriation of several rooms [27]. For example, the plaster room or other day clinic rooms might be used for treating moderately injured patients (yellow), while an intervention room might be used as a second trauma care room, especially for severely injured patients (red). In addition, slightly injured patients might be gathered in the staff dining hall, as illustrated in Figure 1.

However, operating rooms can hardly be misused for treating polytrauma/severe injuries due to hygienic reasons, as outlined in the German S3 guideline [37]. In the investigated hospital, there are currently two operating rooms available exclusively for trauma surgery, which can usually be used quickly for treating severely injured patients in mass casualty incidents or disasters [27]. In addition, there are five operating rooms in the central operating department available (remark: only four rooms can be generally used due to a shortage of staffing). From an organizational point of view, all further operating or intervention rooms available (e.g., rooms for ENT, gynecology, or ophthalmology) are hardly suitable for misuse in the event of a mass casualty incident or disaster.

#### 2.2.3. Instruments and Material

Each of the operating rooms under consideration is designed to be able to care for at least one patient immediately without the need to stock up on consumables. However, if several surgeries take place in a row, this material will have to be refilled for each case. This can be a relevant bottleneck, especially during the night and/or weekends, in the case of longer-lasting events or major disasters. A short-term bottleneck might also be the staff needed for stocking consumables in the operating rooms, and a medium-term bottleneck could be the number of sterile supplies themselves. Sterile cleaning of surgical instruments typically takes about three to four hours [27]. However, in regard to most surgical instruments, more than one set is kept sterile as these resources are also needed for routine surgeries and thus might not always be available sterile because they have just been used. For mass casualty incidents, which usually have a short time horizon, we assumed that those instruments and materials are sufficiently available. Thus, these resources were not modeled for short-term mass casualty incidents.

For disaster planning with a longer time horizon, policymakers have to take into consideration that this resource category should not be underestimated and could be crucial and should, therefore, not be neglected. By investigating several disaster plans, for example, the military and disaster expert Herbert Saurugg [40,41] found for the major Viennese university hospital with about 1700 beds that a crucial bottleneck would be the sterile laundry and scrubs for operating rooms which might be available for just a few days in the case of a longer lasting blackout.

#### 2.2.4. Patient Data

The duration of the treatments and operations, as well as the duration for X-ray and computer tomography examinations, were based on scientific literature, expert interviews, and data from one year in the trauma operating room of this specialized major hospital [27]. In addition, the daily routine workload in the operating rooms was implemented by using simulated real occupancy data for the operating rooms of the trauma department from this hospital for a period of one year. The current construction plan of this hospital was used to model the transportation routes and times. The arrival rates of patients were modeled using exponential distributions.

## 3. Results

In Section 3.1, we simulated different policy scenarios to evaluate the feasibility of the current disaster plan for mass casualty incidents for this acute specialty hospital by identifying bottlenecks and disclosing boundaries:

(1) Impact of the arrival time of injured patients (cf. Section 3.1.1);
(2) Impact of the number of injured patients (cf. Section 3.1.2);
(3) Impact of the number of staff available (cf. Section 3.1.3);
(4) Impact of the number of computer tomography machines available (cf. Section 3.1.4).

The main aim was to identify how many seriously injured patients, together with moderately and slightly injured patients, this hospital could handle properly [27].

Based on these findings, we additionally illustrated for a manageable mass casualty incident scenario how an optimized staff-mix could improve the mean total system time for seriously injured patients using simulation-optimization as well as multiple regression analysis in Section 3.2.

These results provided insight into improving the disaster plan for this hospital and for handling mass casualty incidents in the entire region of Vienna, Austria.

### 3.1. Feasibility of the Current Disaster Plan for Mass Casualty Incidents

First, we simulated the assumptions of the current disaster plan of this acute specialty hospital in a baseline scenario #1 accounting for the following additional emergency patients arriving at 10 p.m. within a time period of 90 min based on exponential distributions:

- Five severely injured patients;
- Twenty moderately injured patients;
- Fifty slightly injured patients.

The following staff-mix was available at the clinic:

- Seventeen medical specialists;
- Five medical assistants;
- Three rotation physicians;
- Twenty general nurses;
- One surgical assistant;
- Four surgical nurses;
- Two radiology assistants.

As the mean waiting time for critical treatment is essential for the survival of severely injured patients, as mentioned before, this outcome measure for policy making was analyzed, and 538 simulation runs were necessary to reach a 95% statistical accuracy of the results for the baseline policy scenario #1 [42]. Table 1 displays the results for 5000 simulation runs of baseline scenario #1, simulating the current disaster plan of the acute specialty hospital.

For the present situation at this hospital, we simulated that the required starting time for a necessary surgery (maximum 720 min from arrival for moderately injured patients) could be met with a 95% statistical accuracy as these patients were operated on average within 336.1 min after arriving at the hospital. Thus, the current disaster plan of this hospital could bear the number of moderately injured patients rather easily.

However, the more important policy parameter of the mean waiting time for critical treatment of severely injured patients showed that the recommended time span between arrival at the hospital and the beginning of their often lifesaving surgery within 120 min could not be met. The mean simulated waiting time for surgery amounted to 168.4 min for severely injured patients. The requirement to start surgery within 120 min after the arrival of severely injured patients at the hospital could only be met in 4.8% of the cases. This result demonstrated that the current disaster plan for this hospital greatly overestimated the number of severely injured patients to be treated in case of a mass casualty incident. This raised the following policy questions:

(1) How many severely injured patients can be realistically treated when prolonging the arrival time (Section 3.1.1)?
(2) How many severely injured patients can be realistically treated at all (Section 3.1.2)?

#### 3.1.1. Impact of the Arrival Time

Next, we investigated the impact of the period of arrival time for emergency patients varying from 45 min (scenario #2), over 120 min (scenario #3), to 200 min (scenario #4) on the mean waiting time for critical treatment compared to the baseline scenario #1 (90 min) as displayed in Table 2 (one-way sensitivity analysis).

**Figure 2:**
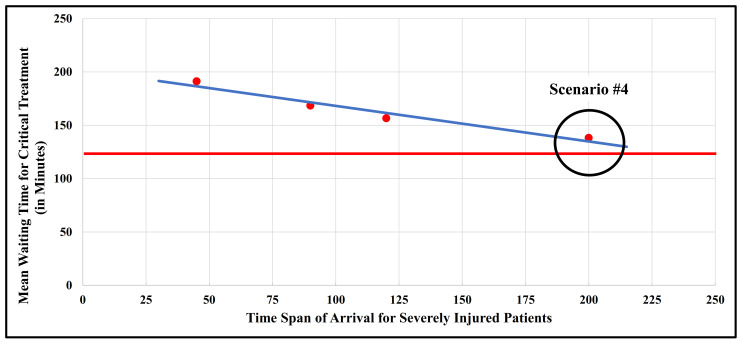
Relationship between the time span of arrival for severely injured patients and their mean waiting time for critical treatment (in minutes) for scenarios #1 to #4. The red dots indicate scenarios #1 to #4 (cf. Table 2), while the blue line is the regression line. The red line displays the maximum mean waiting time for critical treatment of severely injured patients that should be below 120 min. Scenario #4 (red dot) in the black circle is still above this critical time limit (red line).

In scenario #2, the shortening of the time span in which the emergency patients arrive by 50% from 90 to 45 min led to a prolongation of the mean waiting time for critical treatment by 22.9 min for the severely injured patients, amounting to a mean waiting time for critical treatment of 191.3 min. This value exceeded the mean waiting limit for surgery of 120 min by 71.3 min. However, if the emergency patients arrived delayed, such as within 120 min (scenario #3) or 200 min (scenario #4), this would result in a reduction of the mean waiting time for critical treatment of severely injured patients from 168.4 to 156.6 min (scenario #3) and 138.20 min (scenario #4), respectively. The mean waiting time for critical treatment of moderately injured patients for all scenarios (varying between 253.0 and 367.3 min) was below the critical benchmark waiting time for surgery of about 720 min. Therefore, the mean waiting time target for the critical treatment of 120 min regarding severely injured persons would be only met, if the time span of the arrival of the individual patients was higher than 200 min, as illustrated in Figure 2. In such a situation, with prolonged preclinical care, it could be assumed, however, that those emergency patients in need of urgent surgery would no longer arrive at the hospital alive. Those patients who have survived such a long preclinical period are unlikely to require a lifesaving intervention within 120 min. Thus, the current disaster plan was found to be over-optimistic regarding the number of severely injured patients to be treated in this hospital.

#### 3.1.2. Impact of the Number of Patients

To better investigate the emergency patient workload that this specialty hospital could cope with within 90 min (more-way sensitivity analyses), we increased the numbers of emergency patients arriving by +20% (scenario #8), +40% (scenario #9), and decreased these values by −20% (scenario #7), −40% (scenario #6), and −60% (scenario #5) as displayed in Table 3. For example, in the more severe scenario #8, the special acute hospital would be confronted with 6 severely injured, 24 moderately injured, and 60 slightly injured patients.

We were able to show that the time window between the arrival of seriously injured patients and the start of their surgery between 90 min and a maximum of 120 min could only be met with a maximum of approximately three seriously injured patients to be treated (scenario #6), better only two seriously injured patients (scenario #5). The mean waiting time for critical treatment of moderately injured patients varied between 120.4 and 478.4 min for scenarios #5 to #9, respectively, and was found to be non-critical, far below the 720 min limit.

**Figure 3:**
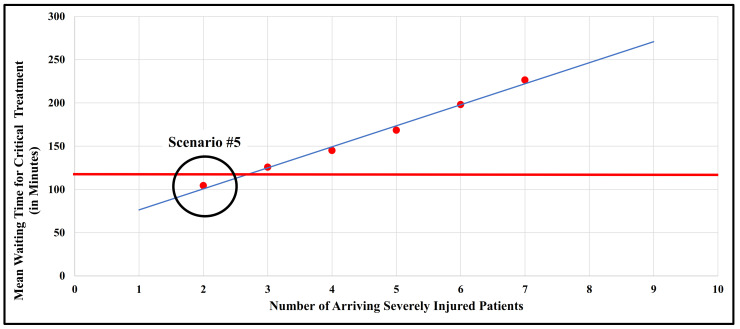
Relationship between the number of arriving severely injured patients and their mean waiting time for critical treatment (in minutes) for scenario #1 and scenarios #5-#9. The red dots indicate scenario #1 and scenarios #5-#9 (cf. Table 3), while the blue line is the regression line. The red line displays the maximum mean waiting time for critical treatment of severely injured patients that should be below 120 min. Scenario #5 (red dot) in the black circle is below this critical time limit (red line).

We revealed a rather linear relationship between the number of seriously injured patients arriving and the mean waiting time for critical treatment, as illustrated in Figure 3. Thus, policymakers could expect an increase in the waiting time for surgery by approximately 20 min per additional seriously injured patient to be treated (scenarios #8 and #9) compared to the baseline scenario #1. This effect reversed the fewer seriously injured patients had to be treated (scenarios #5 to #7). For example, if only two seriously injured patients arrived (scenario #5), then this would further lower the mean waiting time for critical treatment of severely injured patients of baseline scenario #1 from about 168.4 min down to 104.5 min and thus below the limit of 120 min.

#### 3.1.3. Impact of the Number of Staff Available

We then examined the impact of the number of staff available on the key outcome measure of mean waiting time for critical treatment of severely injured patients (one and two-way sensitivity analyses). Due to the extreme shortage of medical staff, we assumed only a realistic increase in these numbers for baseline scenario #1, such as by:

- One additional surgical assistant and one additional radiology assistant (scenario #10);
- One additional rotation physician (scenario #11);
- Two additional rotation physicians (scenario #12).

It was found that the effects on the mean waiting time for critical treatment of severely injured patients could only be marginally influenced by additional staff. For example, the largest change amounted to a reduction of 5.6 min down to 162.8 min by one additional surgical assistant and one additional radiology assistant (scenario #10). Furthermore, one additional rotation physician would only improve the mean waiting time for critical treatment of severely injured patients by 0.8 min to 167.6 min (scenario #11), while two additional rotation physicians would decrease the mean waiting time for critical treatment of severely injured patients by 3.5 min to 164.9 min (scenario #12).

Those improvements by increasing the number of available staff proved to be minor, and the target upper mean waiting time for critical treatment of severely injured patients of 120 min could not be met by scenarios #10 to #12. Please note that because the other medical professions had no bottlenecks and always had enough slacks, we did not investigate increasing those staff categories. To summarize, increasing the number of medical staff was no policy option for this hospital to improve the handling of severely injured patients in mass casualty incidents.

#### 3.1.4. Impact of the Number of Computer Tomography Machines

As computer tomography machines might be a bottleneck for seriously or moderately injured patients (two-way sensitivity analysis), a second computer tomography machine together with a necessary radiology assistant was simulated in scenario #13 compared to only one computer tomography machine in baseline scenario #1.

However, this policy scenario #13 could only change the mean waiting time for critical treatment of seriously injured patients by 5 min to 163.4 min. A similar result was reached in scenario #10 by adding one additional surgical assistant and one additional radiology assistant. To summarize, the policy of increasing the number of computer tomography machines and related staff proved not to be an appropriate measure to reduce the mean waiting time for critical treatment of seriously injured patients to 120 min for this hospital.

### 3.2. Optimizing the Staff-Mix

Next, we investigated an optimal staff-mix for a mass casualty incident baseline scenario #2 (five severely injured patients, 20 moderately injured patients, 50 slightly injured patients, 90 min arrival period) compared to a minimum and a maximum number of staff scenarios for this acute specialty hospital by simulation-optimization as illustrated in Table 4 (more-way sensitivity analyses). As a tradeoff between accuracy level and calculation time, we selected an accuracy level of 99% and could only focus on the mean total system time of severely injured patients in the trauma surgery emergency department as a key policy outcome measure (7000 replications needed) which should be below 200 min in our policy case (i.e., 120 min waiting time to surgery plus an average time for surgery and waiting time for transfer/discharge in our policy case).

An “optimized” minimum number of staff scenario was determined by using the simulation tool Anylogic [29] and the related optimization tool OptQuest on a workstation, which took about 1–2 h.

To understand which of the seven staff categories had the highest impact on the mean total waiting time regarding baseline scenario #2, the baseline staff number was varied from a minimum to a maximum number increased in certain steps, resulting in three scenarios per staff category, as displayed in Table 4. Thus, a total number of 3^7^ = 2187 policy scenarios were enumerated and simulated on a workstation, taking about 3–4 days.

For the minimum number of staff scenario #1, the mean total system time for severely injured patients would amount to 214.37 min compared to 80.29 min in baseline scenario #2. In the maximum number of staff scenario # 2187, the mean total system time for severely injured patients could be reduced to 55.28 min. Then, we investigated which staff-mix scenario would be optimal to guarantee that the outcome measure of the mean total system time for severely injured patients would be lower than 200 min using the OptQuest optimization tool of the simulation software AnyLogic [29]. The “optimized” minimum staff scenario #163 had a mean total system time for severely injured patients of 148.10 min but would assign two more radiology assistants compared to the minimum staff scenario #1 with a mean total system time of severely injured patients amounting to 214.37 min. This OptQuest result was calculated in about 1–2 h and was the second-best result. The global minimum found by enumeration was scenario #82, with a mean total system time for severely injured patients of about 152.86 min, which took about 3–4 days to be calculated.

Next, we analyzed which staff category had the highest impact on the mean total system time for severely injured patients. First, a simple multiple-linear regression analysis was performed on the 2187 enumerated staff-mix policy scenarios to examine the influence of the independent variables medical specialists, medical assistants, rotation physicians, general nurses, surgical assistants, surgical nurses, and radiology assistants on the dependent variable mean total system time for severely injured patients using DATATab [43]. The normality conditions were confirmed:

- Linear graphical relationship of the seven independent staff variables on the dependent variable of the mean total system time for severely injured patients;
- Significant normality of the residuals using four different tests (all four *p*-values < 0.001);
- No multicollinearity of the seven independent staff category variables (all tolerances = 1 thus > 0.1; all variance inflation factors = 1 thus < 10);
- No clear heteroscedasticity of the variance for the residuals.

With an *R*^2^ = 0.7461, the simple multiple-linear regression model showed that the independent variables medical specialists, medical assistants, rotation physicians, general nurses, surgical assistants, surgical nurses, and radiology assistants explained 74.61% of the variance regarding the dependent variable mean total system time for severely injured patients. The model had a standard error of 20.9915 for predicting the mean total system time for severely injured patients. An ANOVA was used to test whether this value deviated significantly from zero (cf. Table 5). Based on the present sample of 2187 staff-mix policy scenarios, we found that the effect significantly deviates from zero: *F* = 914.86, *p* < 0.001, *R*^2^ = 0.7461. As the *R*^2^ (*adjusted*) = 0.7453 and *R*^2^ (*predicted*) = 0.7443, as well as all variance inflation factors (*VIFs*) of the seven independent variables = 1 (no multicollinearity), we obtained a valid simple multiple-linear regression model in the first step.

Thus, the following simple multiple-linear regression model without interaction effects was calculated:

**Mean Total System Time for Severely Injured Patients = 335.88** − 0.71 Medical Specialists − 3.34 Medical Assistants − 9.57 Rotation Physicians − 0.17 General Nurses − 2.98 Surgical Assistants − 8.88 Surgical Nurses − 34.61 Radiology Assistants(1)

**Figure 4:**
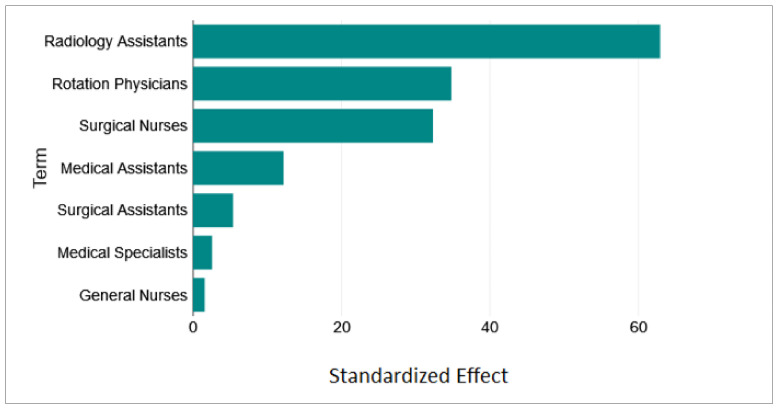
Simple multiple-linear regression model: Pareto chart of the standardized effect on the independent staff category variables on the dependent variable mean total system time for severely injured patients.

Please note that most of the independent staff category variables had a highly significant (*p*-value < 0.001) or high significant (*p*-value < 0.01) impact on the dependent variable mean total system time for severely injured patients, while only the independent variable number of general nurses had no significant impact (*p*-value = 0.12) as shown in Table 5 and in Figure 4, the Pareto chart of the standardized effects. The simple multiple-linear regression analysis showed that increasing the number of radiology assistants would have the highest effect on the mean total system time of severely injured patients (reduction value of −34.61), then increasing the number of rotation physicians (reduction value of −9.57), and also increasing the number of surgical nurses (reduction value of −8.88).

In a second step, we improved the simple multiple-linear regression model by accounting for two-way interactions and quadratic terms by selecting the five most relevant independent variables (*X1*: radiology assistants, *X2*: rotation physicians, *X3*: surgical nurses, *X4*: medical assistants, *X5*: surgical assistants) to fit a suitable multiple-quadratic regression model with two-way interactions to better predict the dependent variable mean total system time for severely injured patients (*Y*) using Minitab [44]. By applying this advanced multiple-regression model, the *R^2^* and the *R^2^* (*adjusted*) of the simple multiple-linear regression could be improved from 0.7446 to 0.9670 and from 0.7453 to 0.9667, respectively. The relationship between the five selected independent variables (*X1* to *X5*) and the dependent (*Y*) variable was highly significant, with *p* < 0.001.

**Figure 5:**
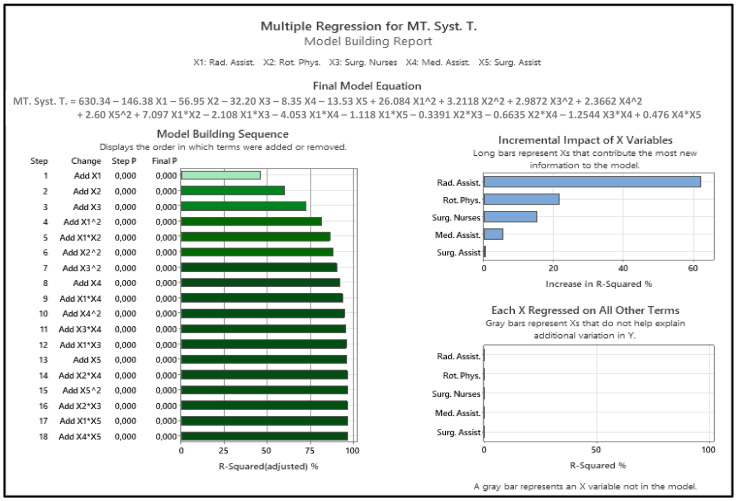
Advanced multiple regression model with two-way interactions and quadratic terms: model building report. Med. Assist. = medical assistants; MT. Syst. T. = mean total system time; Rad. Assist. = radiological assistants; Rot. Phys. = rotation physicians; Surg. Assist. = surgical assistants; Surg. Nurses = surgical nurses.

Figure 5 displays the model-building report for the advanced multiple regression model with two-way interactions and quadratic terms, the incremental impact of the five independent variables (*X1* to *X5*), and the model-building sequence of the equation terms. In the first three building steps of the model, independent variables *X1:* rotation physicians, *X2:* radiology assistants, and *X3:* surgical nurses were added, resulting in an *R^2^* (*adjusted*) = 0.7240. The fourth building step then included a quadratic term (X1*X1: radiology assistants*radiology assistants; *R^2^* (*adjusted*) = 0.8115), while the first two-way interaction term (X1*X2 = radiology assistants*rotation physicians; *R^2^* (*adjusted*) = 0.8633) was added in the fifth building step. With the fourth building step, this advanced model outperformed the simple multiple-linear regression model without interaction effects. With a lower priority, the independent variable *X4:* medical assistants was added in building step 8, while the independent variable *X5:* surgical assistants was included later in building step 13. The last building steps of the model building sequence only marginally improved the results. This further analysis was important to illustrate that three staff categories (*X1:* rotation physicians, *X2*: radiology assistants, *X3:* surgical nurses), including the interaction effect of *X1*: radiology assistants and *X2:* rotation physicians as well as all three quadratic terms of *X1* to *X3* highly impacted the mean total system time for severely injured patients (up to building step 7: *R^2^* (*adjusted*) = 0.9028). As the incremental impact of including *X5:* surgical assistants into the model was neglectable, one might exclude that variable from the model.

Building upon these findings of the above multiple regression analyses, we showed that if the number of radiology assistants was increased by one to two in scenario #82 compared to the baseline scenario #2, then the mean total system time for severely injured patients could be reduced from 214.37 min to 152.86 min (below the 200 min limit), respectively (cf. Table 4). A third radiology assistant on shift in the enumerated minimum staff-mix scenario #163 would then further reduce the mean total system time for severely injured patients to 148.10 min, which was the “optimized” minimum staff-mix scenario #82 by OptQuest.

## 4. Discussion

Based on the results of the simulated and optimized disaster scenarios, policymakers of this acute specialized hospital in Vienna, Austria, were able to understand that the current disaster plan with the treatment requirements for patients of a mass casualty incident could not fully be accomplished with the currently available human resources, structural and constructional resources, as well as equipment for mass casualty incidents.

We showed that especially seriously injured patients would have to wait up to 168.4 min for the start of a lifesaving operation. Assuming that most seriously injured patients would bleed to death after about 120 to 180 min of hospitalization, this waiting time of 168.4 min would not be acceptable [33,45]. Thus, the requirement for five severely injured patients to be treated was too high in the current disaster plan (cf. Section 3.1.2). The treatment of three severely injured patients might be a maximum benchmark (mean waiting time for critical treatment of 125.8 min). In contrast, treatment requirements for moderately injured patients were achievable as their mean waiting time for critical treatment was below the maximum of 720 min. Even if the arrival period of the patients was expanded from 90 to 200 min, the medical requirements would not be met since the mean waiting time for critical treatment of severely injured patients would be 138.2 min (cf. Section 3.1.1).

Subsequently, increasing the number of critical staff (surgical assistants, radiology assistants, rotation physicians) by realistic numbers only led to a marginal improvement in the mean waiting time for critical treatment for severely injured patients (cf. Section 3.1.3). Thus, this would be an inappropriate strategy for policymakers to enhance the current disaster plan. Finally, the policy of increasing the number of computer tomography machines and related staff proved to be not an appropriate measure to reduce the mean waiting time for critical treatment of seriously injured patients down to 120 min (cf. Section 3.1.4).

In a simulation-regression study of 2187 policy disaster scenarios (cf. Section 3.2), we investigated the effect of the staff-mix on the mean total system time for severely injured patients and found that increasing the number of radiology assistants would have the highest effect on the mean total system time of severely injured patients, followed by increasing the number of rotation physicians, and then by increasing the number of surgical nurses.

In summary, the following recommendations for the adaptation of the current disaster plan for this acute specialty hospital can be given:

1. The reduction from five to three seriously injured patients to be treated in the current hospital disaster plan would be the only short-term option to reach the target value of the mean waiting time for critical treatment for these severely injured patients, amounting to 120 min;
2. However, if this reduction is not desired, adequate structural adaptations must be carried out to ensure adequate medical care for more than three seriously injured persons (e.g., a higher number of resuscitation or operating rooms, a higher number of radiology/imaging units) which is a medium and long term project highly dependent on the budgetary situation of this hospital and the political willingness to finance such improvements of the health care supply by policymakers of the City of Vienna.

## 5. Conclusions

In this case study for an acute specialty hospital in Vienna using a discrete-event simulation approach combined with optimization techniques and multiple regression analyses, we found that the number of patients to be treated in a mass casualty incident by the regional disaster plan is unrealistic within a reasonable time. This illustrates the importance of policy modeling for regional decision-makers. Such models can show which targets might be reachable or even disclose those targets that are unrealistic and cannot be met. For example, in this case study, we demonstrated that the treatment policy indicators of the regional disaster plan could only be met by corresponding structural and major staffing adjustments.

Furthermore, policy models should be suitable and realistic for a given case study, accounting for all essential circumstances by applying appropriate methodologies, such as combined methods of simulation, optimization, and multiple regression, as in the case of our study. Often, policy models have limitations that should be considered when applying them to similar related problems requiring different methodological approaches and/or adapted or more comprehensive modeling components.

Limitations of the current study on short-term mass casualty incidents are that we excluded non-critical resource categories that would only become relevant for longer-term mass casualty incidents. For example, the hospitalization of mass casualty patients in general medicine departments or an ICU was not further simulated. Next, for example, instruments and materials were considered as an unlimited and non-critical resource. Then, we only implemented the key and bottleneck staff categories. Finally, additional off-duty or other suitable staff, such as staff on maternity leave or training, former staff members (e.g., people retired, people left), or military/civil servants, were not included due to their not short-term availability. However, for longer-lasting disasters, such as epidemics, bottlenecks regarding the hospitalization of patients, instruments and materials, additional staff categories, and/or additional suitable staff members might be relevant and should be modeled [40,41].

Further research is necessary to create a general model for all suitable hospitals treating trauma patients in mass casualty incidents for the region of Vienna, Austria. Additionally, optimizing the availability of medical staff for acute trauma care in case of mass casualty incidents in this region could be a key strategic target for local policymakers. Furthermore, the interfaces and resources of preclinical and clinical patient treatment should be considered together in order to optimize the scarce overall resources available.

Therefore, in the interest of regional and possibly national disaster planning, policymakers should investigate in detail whether or not the requirements of the underlying disaster plans are realistic and could be achieved with the given resources of hospitals and interlinked caregivers in a region. Suitable Operations Research modeling techniques under consideration of machine learning methods for different disaster policy scenarios could be used for these evaluations.

## Figures and Tables

**Table 1:** Waiting time for critical treatment of patients (in minutes) of the simulated disaster plan for mass casualty incidents in an acute specialty hospital in Vienna, Austria (baseline scenario #1).

	Waiting Time for Critical Treatment Moderately Injured Patients (in Minutes)	Waiting Time for Critical Treatment Severely Injured Patients (in Minutes)
**Mean**	336.1 (720)	168.4 (120)
**Standard Deviation**	119.5	41.6
**Median**	336.6	165.2
**Minimum**	38.6	47.2
**Maximum**	747.6	322.8
**Range**	709	275.6

Maximum upper value for the mean waiting time for critical treatment is listed in parentheses.

**Table 2:** Impact of the arrival period for emergency patients on the waiting time for critical treatment (in minutes) of the simulated disaster plan for mass casualty incidents in an acute specialty hospital in Vienna, Austria (baseline scenario #1) compared to a more severe scenario #2 and two less severe scenarios #3 and #4.

		Baseline Scenario #1	Scenario #2	Scenario #3	Scenario #4
**Policy Variables**	**Period of Arrival for Emergency Patients** **(in minutes)**	90	45	120	200
	**Number of Severely Injured Patients**	5	5	5	5
	**Number of Moderately Injured Patients**	20	20	20	20
	**Number of Slightly Injured Patients**	50	50	50	50
**Waiting Time for Critical Treatment of** **Severely Injured Patients** **(in Minutes)**	**Mean**	**168.4**	**191.3 (120)**	**156.6 (120)**	**138.2 (120)**
	**Standard Deviation**	41.6	44.3	40.9	39.1
	**Median**	165.2	189.4	153	134.2
	**Minimum**	47.2	56.6	49	46.9
	**Maximum**	168.4	191.3	156.6	314.6
	**Range**	275.6	56.6	243.8	267.7
**Waiting Time for Critical Treatment of** **Moderately Injured Patients** **(in Minutes)**	**Mean**	**336.1 (720)**	**367.3 (720)**	**290.6 (720)**	**253.0 (720)**
	**Standard Deviation**	119.5	121	121.6	123.7
	**Median**	336.6	366.8	294.9	243.6
	**Minimum**	38.6	43.7	56.9	45.2
	**Maximum**	747.6	809	657.5	591.9
	**Range**	709	765.3	600.6	546.6

Maximum upper value for the mean waiting time for critical treatment is listed in parentheses.

**Table 3:** Impact of the number of severely injured patients on the waiting time for critical treatment of patients (in minutes) of the simulated disaster plan for mass casualty incidents in an acute specialty hospital in Vienna, Austria (baseline scenario #1) compared to two more severe scenarios #8 and #9 and three less severe scenarios #5, #6, and #7.

		Baseline Scenario #1	Scenario #5	Scenario #6	Scenario #7	Scenario #8	Scenario #9
**Policy Variables**	**Period of Arrival for** **Emergency Patients** **(in minutes)**	90	90	90	90	90	90
	**Number of Severely Injured Patients**	5	2	3	4	6	7
	**Number of Moderately Injured Patients**	20	8	12	16	24	29
	**Number of Slightly Injured Patients**	50	20	30	40	60	72
**Waiting Time for Critical Treatment of** **Severely Injured Patients** **(in Minutes)**	**Mean**	**168.4**	**104.5**	**125.8**	**144.9**	**198.1**	**226.5**
	**Standard Deviation**	41.6	34.7	37.7	38.7	43	42.3
	**Median**	165.2	101.5	121.3	141.6	196.1	222
	**Minimum**	47.2	38.2	42.8	50.2	89.4	107.5
	**Maximum**	322.8	294.8	296.2	287.8	356.6	399.9
	**Range**	275.6	256.6	253.3	237.6	267.2	292.4
**Waiting Time for Critical Treatment of** **Moderately Injured Patients** **(in Minutes)**	**Mean**	**336.1**	**120.4**	**204.1**	**282.1**	**391**	**478.4**
	**Standard Deviation**	119.5	76.7	103.2	116.5	135	131.3
	**Median**	336.6	92.7	192.4	286.3	383.2	475
	**Minimum**	38.6	36.6	37.2	47.8	56.5	52.2
	**Maximum**	747.6	441.1	539.2	695.9	831.6	843.6
	**Range**	709	404.5	502	648.1	775.1	791.4

Maximum upper value for the mean waiting time for critical treatment was 120 min for severely injured patients and 720 min for moderately injured patients, respectively.

**Table 4:** Simulation-optimization and enumeration of the optimum staff-mix for baseline mass casualty incident scenario #2.

Staff Category	Number of Staff in Baseline Scenario #2	Minimum Number of Staff in Scenario #1	Maximum Number of Staff in Scenario #2187	Enumerated Minimum Number of Staff in Scenario #82	“Optimized” Minimum Number of Staff in Scenario #163	Increase Steps of the Number of Staff for Scenarios (Number of Staff)	Number of Scenarios
**Medical Specialists**	11	8	12	8	8	2 (8, 10, 12)	3
**Medical Assistants**	5	3	7	3	3	2 (3, 5, 7)	3
**Rotation Physicians**	6	4	8	4	4	2 (4, 6, 8)	3
**General Nurses**	23	15	25	15	15	5 (15, 20, 25)	3
**Surgical Assistants**	2	1	3	1	1	1 (1, 2, 3)	3
**Surgical Nurses**	5	4	8	4	4	2 (4, 6, 8)	3
**Radiology Assistants**	2	1	3	2	3	1 (1, 2, 3)	3
**Mean Total System Time for Severely Injured Patients** **(in Minutes)**	**80.29**	**214.37**	**55.28**	**152.86**	**148.1**		

Maximum mean total system time of severely injured patients (bold line at the bottom) in the trauma surgery emergency department should be below 200 min.

**Table 5:** Simple multiple-linear regression model: optimal staff-mix of baseline mass casualty incident baseline scenario #2.

Modell	Un-Standardized Coefficients	Standardized Coefficients	*Standard Error* * Coefficient*	*t*	*p*	95% Confidence Interval for *B*	
	*B*	*Beta*				Upper Bound	Lower Bound
**(Constant)**	335.88		4.73	71.06	<0.001	345.15	326.6
**Medical Specialists**	−0.71	−0.03	0.27	−2.58	0.01	−0.17	−1.25
**Medical Assistants**	−3.34	−0.13	0.27	−12.17	<0.001	−2.81	−3.88
**Rotation Physicians**	−9.57	−0.38	0.27	−34.81	<0.001	−9.03	−10.11
**General Nurses**	−0.17	−0.02	0.11	−1.56	0.12	0.04	−0.39
**Surgical Assistants**	−2.98	−0.06	0.55	−5.43	<0.001	−1.9	−4.06
**Surgical Nurses**	−8.88	−0.35	0.27	−32.3	<0.001	−8.34	−9.42
**Radiology Assistants**	−34.61	−0.68	0.55	−62.95	<0.001	−33.53	−35.68

## Data Availability

The main data that support the findings of this study are available in Staribacher [27]. Further data are restricted by the acute specialty hospital and are therefore not publicly available.
